# Supplementary material for: Effects of Vine Tea Extract on Meat Quality, Gut Microbiota and Metabolome of Wenchang Broiler
Source: Animals (Basel). 2022 Jun 28;12(13):1661. doi: 10.3390/ani12131661 (PMC9265100; doi:10.3390/ani12131661)
Supplement: Supplementary file 1 [file animals-12-01661-s001.zip › animals-1751172-supplementary.pdf]

Supplementary materials

Table S1 Alpha diversity indexes inter-group difference table.

Item	Groups		SEM	*P*
	T1	T3		
observed_species	716.00	748.20	32.54	0.504
shannon	6.95	6.82	0.17	0.626
simpson	0.98	0.97	0.01	0.227
chao1	821.38	901.17	43.31	0.229
ACE	830.98	897.99	38.63	0.255
goods_coverage	99.52	99.42	0.04	0.143

T1: the broilers were fed a basal diet; T3: the broilers were fed a basal diet supplemented with AGE at 0.4%.

Table S2 Differential metabolites identified among T1 and T3 groups from the data set of the feces samples

No.	Differential metabolites	Score[1]	RT (min)	Formula	*P*-Value[2]	Fold change[2]
1	p-cresol sulfate	0.48	0.402	$C_7H_8O_4S$	0.018	0.22
2	cholesterol sulfate	0.66	0.405	$C_{27}H_{46}O_4S$	0.049	0.53

1) The values were the degree of secondary spectrum (MS^2) matching between the analytical compounds of base peak intensity chromatogram and an in-house MS^2 database (Biotree DB, Shanghai, China) in positive ion mode. The property of the value is between [0,1], and the larger the value, the higher the matching.

2) Upregulation ($p < 0.05$, FC > 1.10); Downregulation ($p < 0.05$, FC < 0.90).

Table S3 MS data and identification of dihydromyricetin metabolites in broiler feces.

Name	RT (min)	Ion mode[1]	Metabolic pathway	Formula	Fragment ions
M1	1.004	[M-H]	Dehydration, Reduction	$C_{15}H_{12}O_7$	125.02383,151.03963,177.01920
M2	3.174	[M-H]	Dehydration, Nitro Reduction, Glycine Conjugation	$C_{17}H_{15}NO_6$	190.01439,166.04994,117.03430
M3	3.409	[M-H]	Nitro Reduction, Nitro Reduction, Acetylation	$C_{17}H_{18}O_5$	257.11786，185.09554,93.03414

Table S4 MS data and identification of myricetin metabolites in broiler feces.

Name	RT(min)	Ion mode[1]	Metabolic pathway	Formula	Fragment ions
M1	1.004	[M-H]	Nitro reduction	$C_{15}H_{12}O_7$	125.02383,151.03963,178.99844
M4	3.636	[M-H]	oxidation Nitro reduction, methylation	$C_{16}H_{12}O_6$	255.06613,135.00806,119.04892
M5	0.871	[M+H]	Nitro reduction, Nitro reduction, methylation	$C_{16}H_{16}O_4$	229.08534,147.04385

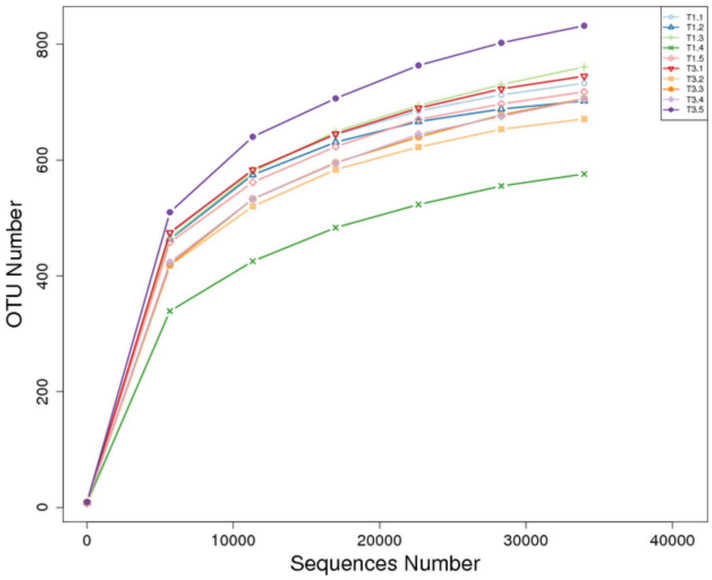

Figure S1 Dilution curves

T1: the broilers were fed a basal diet; T3: the broilers were fed a basal diet supplemented with AGE at 0.4%.

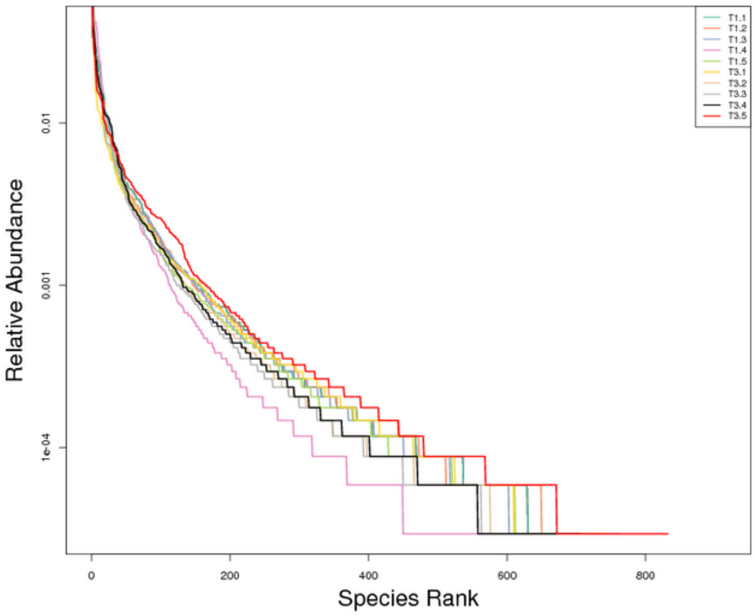

Figure S2 Rank abundance curves

T1: the broilers were fed a basal diet; T3: the broilers were fed a basal diet supplemented with AGE at 0.4%.

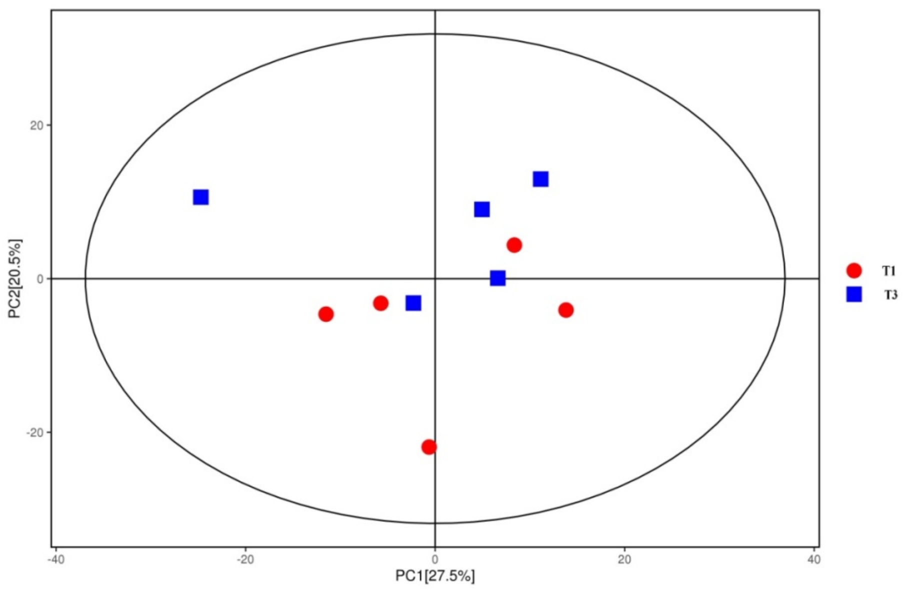

Figure S3 PCA score plot.

T1: the broilers were fed a basal diet; T3: the broilers were fed a basal diet supplemented with AGE at 0.4%.

A PLS-DA scores

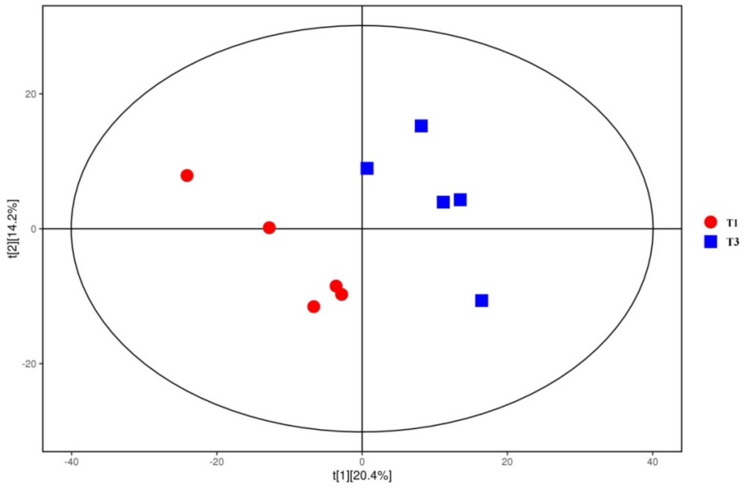

B PLS-DA permulation

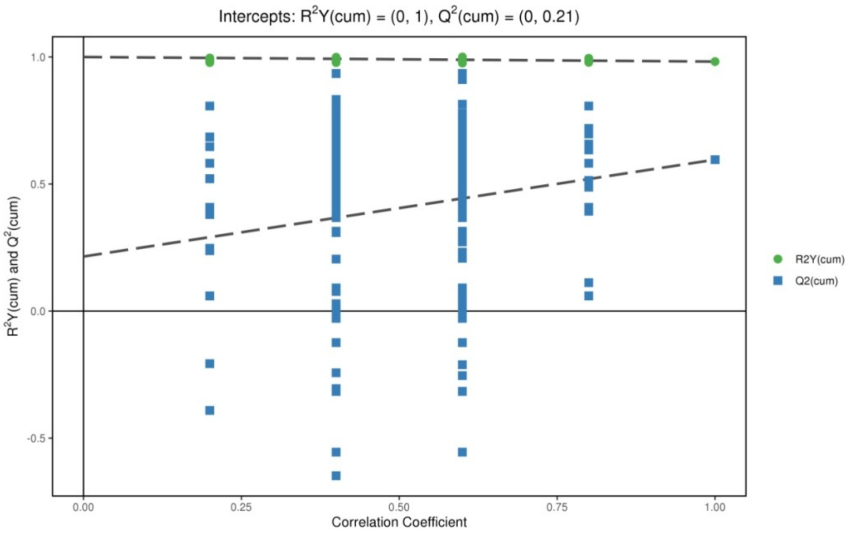

Figure S4 PLS-DA analysis.

A) PLS-DA scores: The abscissa represents the first principal component PC1, and the ordinate represents the second principal component PC2. T1: the broilers were fed a basal diet; T3: the broilers were fed a basal diet supplemented with AGE at 0.4%.

B) Permulation diagram: R2 stands for model verification, and the Y matrix of original classification and *N* times of different arrangement are linearly regressed with R2Y and Q2Y, and the intercept values of regression line and y-axis are R2 and Q2, respectively; used to measure whether the model is over-fitted.
